# The Importance of Bank Vole Density and Rainy Winters in Predicting Nephropathia Epidemica Incidence in Northern Sweden

**DOI:** 10.1371/journal.pone.0111663

**Published:** 2014-11-12

**Authors:** Hussein Khalil, Gert Olsson, Frauke Ecke, Magnus Evander, Marika Hjertqvist, Magnus Magnusson, Mikaell Ottosson Löfvenius, Birger Hörnfeldt

**Affiliations:** 1 Department of Wildlife, Fish, and Environmental Studies, Swedish University of Agricultural Sciences, Umeå, Sweden; 2 Department of Aquatic Sciences and Assessment, Swedish University of Agricultural Sciences, Uppsala, Sweden; 3 Department of Clinical Microbiology, Division of Virology, Umeå University, Umeå, Sweden; 4 Swedish Institute for Infectious Disease Control, Stockholm, Sweden; 5 Department of Forest Ecology and Management, Swedish University of Agricultural Sciences, Umeå, Sweden; University of California, San Francisco, United States of America

## Abstract

Pathogenic hantaviruses (family *Bunyaviridae*, genus *Hantavirus*) are rodent-borne viruses causing hemorrhagic fever with renal syndrome (HFRS) in Eurasia. In Europe, there are more than 10,000 yearly cases of nephropathia epidemica (NE), a mild form of HFRS caused by Puumala virus (PUUV). The common and widely distributed bank vole (*Myodes glareolus*) is the host of PUUV. In this study, we aim to explain and predict NE incidence in boreal Sweden using bank vole densities. We tested whether the number of rainy days in winter contributed to variation in NE incidence. We forecast NE incidence in July 2013–June 2014 using projected autumn vole density, and then considering two climatic scenarios: 1) rain-free winter and 2) winter with many rainy days. Autumn vole density was a strong explanatory variable of NE incidence in boreal Sweden in 1990–2012 (R^2^ = 79%, p<0.001). Adding the number of rainy winter days improved the model (R^2^ = 84%, p<0.05). We report for the first time that risk of NE is higher in winters with many rainy days. Rain on snow and ground icing may block vole access to subnivean space. Seeking refuge from adverse conditions and shelter from predators, voles may infest buildings, increasing infection risk. In a rainy winter scenario, we predicted 812 NE cases in boreal Sweden, triple the number of cases predicted in a rain-free winter in 2013/2014. Our model enables identification of high risk years when preparedness in the public health sector is crucial, as a rainy winter would accentuate risk.

## Introduction

Zoonotic diseases are diseases transmitted from vertebrate animal hosts to humans. They constitute more than half of known human pathogens [1] and incur severe health and economic costs on societies [2]. Anthropogenic expansion and land use modification has contributed to the emergence and re-emergence of several zoonoses [3]. To curb the incidence of zoonotic diseases, researchers have attempted to identify spatial and temporal patterns in infection risk, e.g. [4]–[7]. Predicting the likelihood of zoonotic-disease outbreaks under different ecological settings enables implementing adequate measures to raise public awareness, preparedness, and ultimately disease prevention.

Pathogenic hantaviruses (family *Bunyaviridae*, genus *Hantavirus*) are rodent-borne RNA viruses and etiologic agents of hantavirus pulmonary syndrome in the Americas and hemorrhagic fever with renal syndrome (HFRS) in Eurasia [8]–[9]. Human infections mostly occur through inhalation of viral particles secreted or excreted by infected rodents. In Europe, there are more than 10 000 cases of HFRS annually; most of which are nephropathia epidemica (NE), a mild form of HFRS caused by Puumala virus (PUUV) [10]–[11]. Mortality due to NE is low, but morbidity may be high especially in the event of renal impairment [12]–[13].

The bank vole (*Myodes glareolus*) is the host of PUUV [14]. It is common and widely distributed across Europe [15] yet exhibits different dynamics across its range. In the boreal zone of Fennoscandia, bank vole populations undergo 3 to 4 year cycles, e.g. [16]–[18]. Bank vole distribution in the landscape expands and contracts in accordance with the phase of the cycle [18].

However, in Western and Central Europe, cyclic fluctuations in bank vole densities are generally missing (but see [19]), and seasonal and inter-annual variation in vole density partly depend upon high production of beech and oak seeds (mast years) [20].

NE incidence is positively related to bank vole density [4], [21]–[24] and in the temperate zone related to environmental factors driving bank vole outbreaks, e.g. high summer and autumn temperatures two and one year earlier, respectively [25]–[26]. Human exposure to PUUV is further modified by the behavior of humans and bank voles [21], [22]. In Belgium, NE incidence peaks early in summer due to the increase in human outdoor activities, especially as bank vole densities are high in summer [27]. However, in Fennoscandia, bank voles most often reach their annual population peak in autumn after the conclusion of the reproductive season, and human incidence is highest during winter months, when bank voles infest buildings [28]–[29]. Hence, although Northern Fennoscandia and Western and Central Europe share the same virus-host system, region-specific host ecology gives rise to distinct patterns in NE incidence [10].

Climate may affect frequency and spatial scale of Hantavirus outbreaks through its influence on host populations, e.g. an increase in the frequency of weather driven mast years (years with high beech and oak seed production) in Western Europe may be associated with increased frequency of NE outbreaks [30]. In northern latitudes snow cover provides small mammals with shelter from predators [16], access to insulation [31], and access to food in the subnivean space [32]. Hence, weather conditions affecting snow properties such as its thickness, structure, density, and thermal properties, may affect bank vole winter survival and behavior. Hence, if weather conditions during winter are unfavorable, bank voles may move into buildings for protection against adverse weather [29] and shelter from predators, further increasing human exposure to excreted PUUV [21], [23], [33].

**Figure 1:**
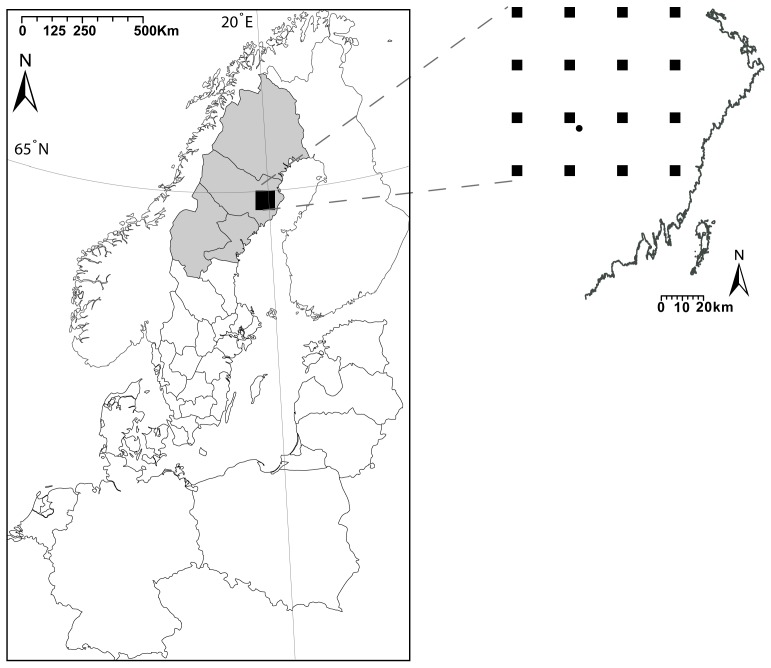
Map of the study area. The black square represents the 100 ×100 km rodent monitoring area. The grey area constitutes the four northernmost counties in Sweden, representing 55% of the total area of Sweden, and used to calculate and predict incidence. The blow-up shows the trapping design and the location of Svartberget climate station (black circle), from which temperature and precipitation data to classify rainy winter days were retrieved.

In Sweden, NE has been a notifiable disease since 1989. More than 90% of cases occur in the four northernmost counties [22], [34], covering 55% of the Swedish surface area (Fig. 1). Between July and June of years 2006/2007 and 2007/2008, there were outbreaks of NE that led to record numbers of reported cases; 1394 and 1481, respectively. The outbreak NE season of 2006/2007 occurred during an increase phase of the bank vole population and coincided with a mild winter. There were several reports that more voles than usual were trapped inside buildings [12]. The outbreak season of 2007/2008 coincided with peak rodent density in autumn 2007- the highest since 1973 [23]. Prior to 2006/2007, the largest NE outbreak was reported in 1998–1999 (589 cases) and also coincided with peak vole density in autumn. Olsson et al. [23] demonstrated, using a simple yet adequate model, the strong connection between bank vole density in autumn and NE incidence. Autumn vole density explained more than 70% of the variation in NE incidence (cf. [23]).

In this study, we built on the model introduced by Olsson et al. [23] to explain and forecast NE incidence using bank vole densities. Weather conditions during winter may modify small mammal survival [23] and movement; most likely including their propensity to enter building [29]. Specifically, we tested whether the number of rainy days in winter, in addition to bank vole density, contributed to the variation in NE incidence. Furthermore, as the proportion of landscape occupied by bank voles varies among phases of the vole cycle [18], we evaluated whether the strength of association between NE incidence and bank vole density also differs between phases. In years when vole distribution in the landscape is limited, typically during low-density phases, disease risk associated with changes in vole density may be local. Vole density variation during such years may thus contribute less to inter-annual variation of NE incidence. Hence, in addition to confirming NE incidence pattern in relation to bank vole density, we tried to infer mechanisms contributing to the aforementioned pattern and consequently, refine our predictive model without sacrificing its simplicity. We finally predicted NE incidence in 2013–2014 using projected bank vole density only, and then considering two climatic scenarios: 1) a winter with numerous rainy days similar to winter 2006/2007 and 2) a rain-free winter.

## Methods

### Ethics statement

Permission to trap small mammals have been obtained from the Swedish. Environmental Protection Agency (SEPA; latest permission: Dnr 412-4009-10) and from the Animal Ethics Committee in Umeå (latest permission: Dnr A-61-11).

### Nephropathia epidemica data

We used NE incidence data in 1990–2012 for all subsequent analyses, excluding NE season of 1989/1990. NE became a notifiable disease in 1989, and we considered the first year to be an initiation year with underestimated incidence. Preliminary analyses showed that NE season 1989/1990 was a negative outlier, yet including it did not significantly alter any of our results. Most cases of NE in Sweden occur during late fall and in winter, with incidence significantly related to bank vole density [22], [23]. We used reported number of NE cases to calculate incidence in Northern Sweden. More than 90% of yearly NE cases in Sweden are reported from the four northernmost counties of Jämtland, Västernorrland, Västerbotten, and Norrbotten, and cases reported elsewhere mostly pertain to NE infections acquired while residents were on vacation in the north [23]. We sorted data into “NE seasons” by pooling cases from July year *t* through June year *t+1*. For example, NE season 2006/2007 represents the sum of NE cases in July 2006–June 2007.

### Bank vole data

Data on bank vole densities were available through the National Environmental Monitoring Program, run by the Swedish Environmental Protection Agency; and initiated as a research project in 1971 in the region of Västerbotten, Northern Sweden [17], . Within the 100 × 100 km small rodent monitoring area (Fig. 1), snap trapping of rodents takes place twice a year (spring and autumn) in 58 1-ha plots. Detailed description of trapping methods and sampling design are given elsewhere [17], [18]. We used autumn bank vole trapping indices (hereafter referred to as density), calculated as the number of bank voles trapped per 100 trap-nights, to explain NE incidence. Additionally, to forecast NE incidence in 2013/2014 several months before the bulk of NE infections occur, we multiplied observed bank vole density in spring2013 by expected vole population growth rate in summer to predict autumn vole density. Projected autumn bank vole density  =  bank vole density in spring × Expected growth rate in summer

Bank vole population summer growth rate is phase-dependent Population growth rate is highest during the increase phase of the cycle and declines in subsequent phases [17], [18]. In 2013, bank vole population was in the increase phase of the cycle. Using bank vole trapping data since the initiation of small mammal monitoring in 1971, vole population growth during summer 2013 was calculated as median growth rate in the increase phase of the cycle. Additionally, we considered a bold estimate of summer growth rate (1 unit higher than median growth rate) and a conservative estimate (1 unit lower) to allow for uncertainty in summer population growth rate in 2013.

We classified autumn vole densities in 1971–2012 into either low or high density years based on the phase of the vole cycle. The transition between successive cycles is characterized by a major shift in rate of change from low to high values in the reproduction season of the increase phase, yr 1 [17], [18]. Subsequent phases were numbered sequentially as yr 2, yr 3 and sometimes yr 4. Yr 1 and 2 represented the increase and peak phases and were classified as high density years, whereas yr 3 and 4 represented decrease and low years and were classified as low density years. To evaluate the consequences of dynamic vole distribution on the accuracy of predicting NE incidence, we calculated bank vole landscape occupancy as the proportion of plots (total n = 58) where at least 1 bank vole was trapped in autumn.

### Meteorological data

Weather and snow conditions during winter may affect the behavior of bank voles and other small mammals, and drive them into peridomestic buildings, e.g. outhouses, wood sheds, and human dwellings [12], [23], [29], [33]. Snow conditions in mild winters in Fennoscandia may become unfavorable upon rainfall through ground icing [31], [35], which amongst others may prevent small mammals, including bank voles, from accessing underground sites for insulation and protection from predators.

We retrieved diurnal air temperature and precipitation from the reference climate station at Svartberget Forest in Vindeln, located within the rodent monitoring area (Fig 1). Following Hansen et al. [35], we calculated the number of rainy days from December to March in each year. Days were classified as rainy if the following conditions were met: 1) average diurnal temperature was higher than 0°C and 2) more than 1 mm precipitation was recorded. Hansen et al. [35] included days with average temperature higher than 1°C rather than 0°C. Nevertheless, we consider precipitation during days with above 0°C average temperature would most likely enhance metamorphosis of snow and trigger bank vole infestation of buildings. Consequently, in addition to bank vole density, we used the number of rainy days from December to March (hereafter referred to as winter) to explain the variation in incidence and predict the number of cases in the NE season of 2013/2014.

### Statistical analysis

To update the pattern reported by Olsson et al. [23], we fitted a univariate linear regression model to explain the variation in NE incidence in 1990–2012 with observed bank vole density in autumn as an explanatory variable. Using the regression equation of the updated model and projected bank vole densities in autumn 2013 (predicted from observed spring 2013 density, see above), we forecasted incidence in the NE season of 2013/2014.

We also related autumn bank vole density in 1971–2012 to its landscape occupancy. The aim was to verify that high density years were characterized by a wider bank vole distribution. Subsequently, we fitted two separate univariate linear regression models to explain NE incidence, one during early years of the cycle (high density) and another during later (low density) years of the cycle. We compared the fit of the two models to determine whether the accuracy of predicting NE incidence differed between earlier and later years of the cycle.

We investigated the influence of winter conditions on incidence in NE seasons 1990–2012 by fitting a multiple regression model using observed autumn bank vole density and number of rainy days in winter as predictors. Additionally, to confirm that NE incidence is genuinely related to the number of rainy days in winter and not simply to warmer winters, we fitted a second model using observed bank vole autumn density and number of winter days with average temperature higher than 0°C as predictors. We compared the fit of the models using AIC values.

Finally, we used the resulting regression equation to forecast incidence based on projected bank vole density in autumn 2013 and two climatic scenarios. In the first scenario, we predicted the number of NE cases in a mild 2013/2014 winter with 13 rainy days, equaling the highest number of rainy days throughout the study period, occurring during NE season 2006/2007. For the second scenario, we considered a rain free winter and thus 0 rainy days, which occurred during NE season 2012/2013.

In all analyses, we normalized NE incidence data through natural log transformation. Bank vole data (both density and landscape occupancy) represented proportions and were arcsine transformed. We also checked for normality of residuals and for highly influential points in all models. Analyses were carried out in the statistical software R [36]. All regression models were fitted through the basic R package using ordinary least squares method, and statistical significance was considered to be reached at a probability of less than 0.05.

## Results

### Explaining NE incidence

**Figure 2:**
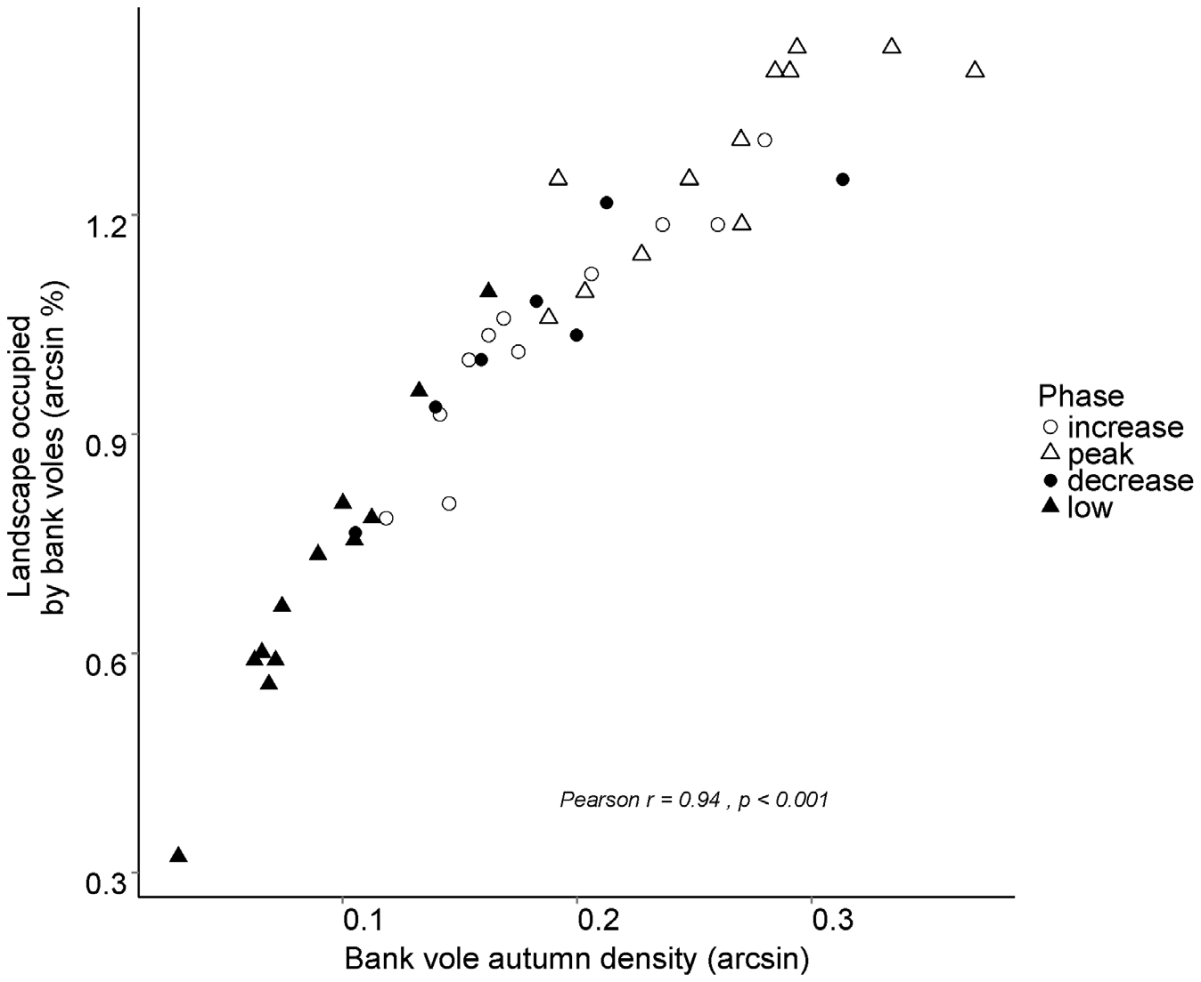
The relationship between proportion of occupied 1-ha plots (arcsine-transformed) (total number  = 58) and autumn bank vole density (number of trapped individuals per 100 trap nights) (arcsine-transformed) in 1971–2012 during the four phases of the vole cycle.

**Figure 3:**
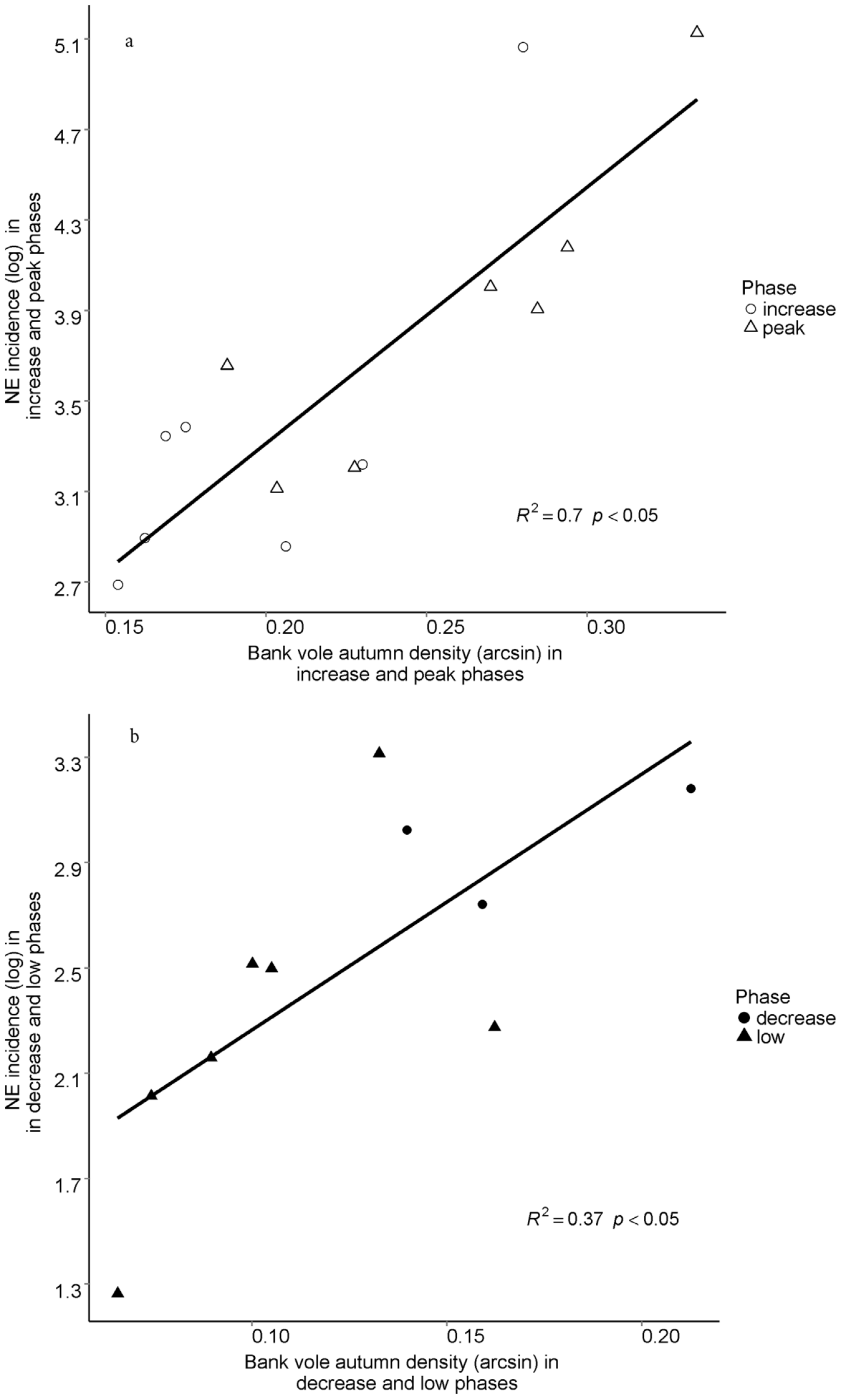
Relationship between annual (July–June) nephropathia epidemica incidence (NE) (log-transformed) in Northern Sweden and bank vole autumn density (number of trapped individuals per 100 trap nights) (arcsine transformed) during a) increase and peak years (n = 13) and b) decrease and low years (n = 11) of the vole cycles in 1990–2012.

Autumn bank vole density was significant in explaining NE incidence in Northern Sweden in 1990–2012 (p<0.001, df = 21) and explained 79% of its variation. The regression equation (1) was:
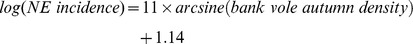



Bank vole landscape occupancy in 1971–2012 increased with density (Pearson correlation, r = 0.94, p<0.001, df = 40, Fig.2). Mean occupancy during high-density years was 82%, significantly higher than the 54% mean occupancy during low-density years (t-test, df = 29, p<0.001). Bank vole autumn densities in both low and high years in 1990–2012 were significantly related to NE incidence (p<0.05 and p<0.001 for low and high density years, respectively); however, during low density years only 37% of variation in NE incidence was explained, compared to 71% in high density years (Fig 3). The standard error around the slope was higher for the low density years model (slope (SE): 10.3 (3.9)) compared to the high density years model (slope (SE): 11.4 (2.1)).

**Figure 4:**
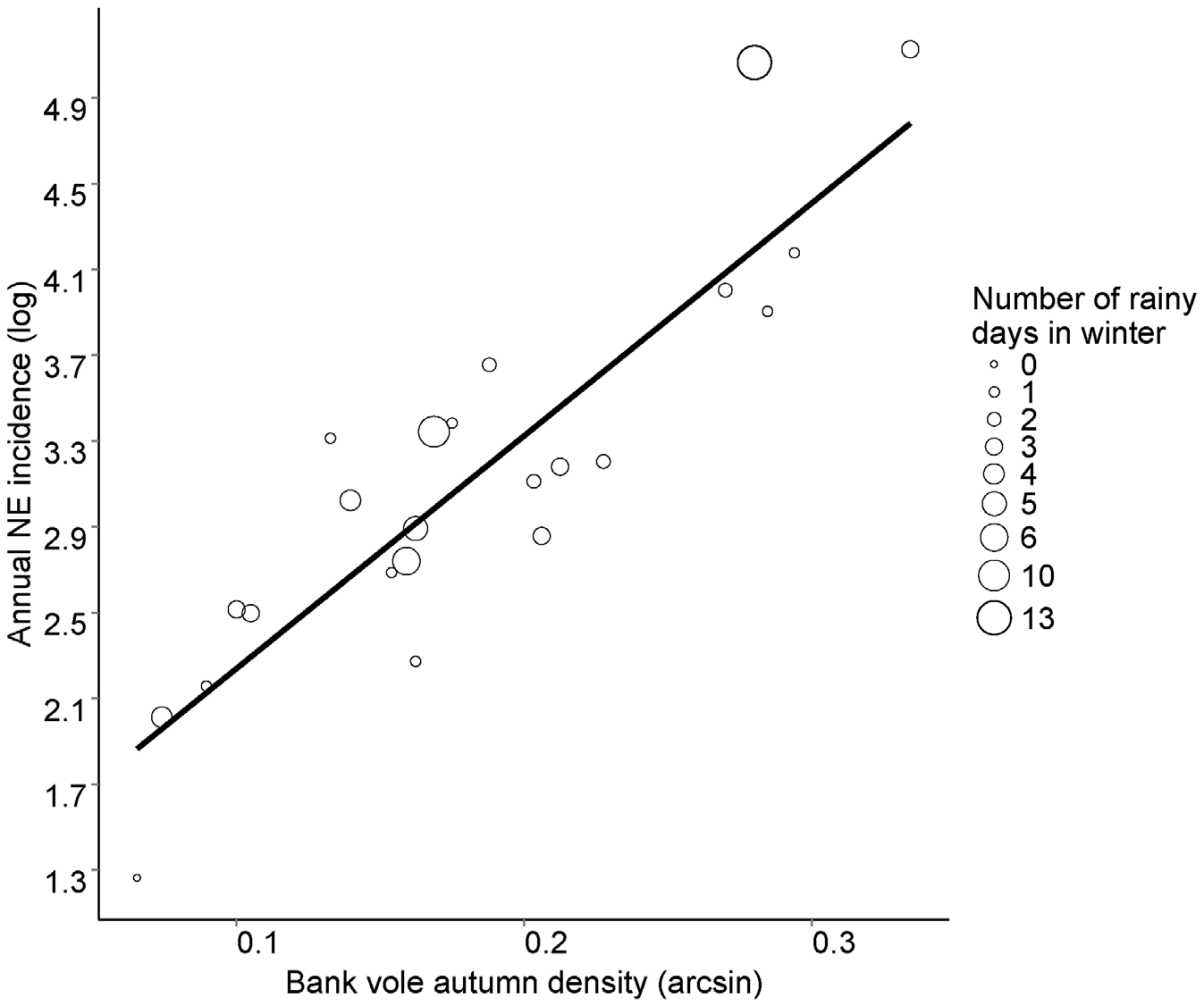
Relationship between annual (July–June) nephropathia epidemica (NE) incidence (log-transformed) in N. Sweden and bank vole autumn density in 1990–2012 (arcsine transformed). The size of circles is proportional to the number of rainy days in winter. Both vole density and number of rainy days were significant predictors of NE incidence (p<0.001 and p<0.05, respectively, n = 24).

Adding the number of rainy days in winter to the model explaining NE incidence in 1990–2012 improved the model significantly (Fig. 4). The improved model explained 84% of the variation in yearly NE incidence. AIC of the simpler model with only autumn bank vole density was 28.9 and dropped to 24.3 upon incorporating the number of rainy days in winter. Both variables were significant (p<0.001 and p<0.05, respectively). The regression equation (2) with two predictor variables was:
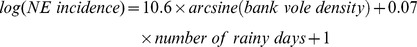



The number of rainy days and the number of days with average temperature above 0°C were expectedly correlated (Spearman correlation, p<0.05). To test whether substituting the number of rainy days with the number of days with average temperature above 0°C would significantly explain NE incidence, we fitted a linear model using autumn bank vole density and number of days with average temperature above 0°C as predictors. However, this did not improve model fit. The model explained 81% of the variation, yet the AIC value increased from 24.3 to 27.7; and only bank vole density in autumn was significant despite number of days with temperature above 0°C showing a trend.

### Predicting NE incidence in July 2013–June 2014

To forecast NE incidence in the NE season 2013/14, we first projected bank vole density in autumn 2013 through multiplying observed spring 2013 vole density (0.40 bank voles per 100 trap nights) by expected growth rate in the intervening summer. Median summer growth rate during an increase year was 12.5, and accordingly; bank vole density in autumn was predicted to be 5 per 100 trap nights. A conservative summer growth rate of 11.5 and a bold summer growth rate of 13.5 would yield autumn bank vole densities of 4.6 and 5.4 per 100 traps nights, respectively. Using the regression equation (1) above and density of five bank voles per 100 trap nights, we forecast the number of NE cases in Northern Sweden to be 326; an incidence of 37.1. A more conservative prediction based on bank vole density of 4.6 per 100 trap nights yielded an estimate of 295 cases, an incidence of 33.5. A bolder bank vole density of 5.4 per 100 trap nights, however, resulted in an estimate of 361 cases, an incidence of 41.1.

Forecasting NE incidence using projected bank vole density of five per 100 trap nights in autumn 2013 given two climatic scenarios yielded disparate outcomes. Using the regression equation (2) of the multivariate model; in a mild winter scenario with 13 rainy days, we forecast 812 cases in 2013, an incidence of 92 in Northern Sweden. However, in a rain free winter, the number of expected cases would be less: 260, an incidence of 29.6.

## Discussion

Identifying areas or time periods of high disease risk is challenging due to the underlying dynamic processes as vertebrate reservoirs abundance and distribution varies spatially and temporally [37], Bank vole autumn density is a strong predictor of NE incidence [4], [23] in boreal Sweden, as has been also shown in Finland [21]. We here report for the first time a model supporting the hypothesis that risk of NE outbreak is higher in rainy winters.

Direct, mostly airborne, transmission of PUUV to humans may in part account for the strong association between host density and NE incidence [21], [24]. As PUUV has one competent host and does not depend on an arthropod vector for transmission, the ecological link between bank vole density and NE incidence is not modified by vector dynamics and distribution. On the other hand, the relationship between host density of vector-borne pathogens, e.g. Lyme disease and Tularemia, and human incidence depends on vector ecology and distribution [38].

Distribution of bank voles in the landscape changes within cycles [17], [18]. During low and decrease phases of the cycle, bank voles were present only in a fraction of the landscape they occupied during increase and peak phases (Figure 2). Indeed, we found that NE incidence was more accurately predicted during high-density years (Figure 3). As bank vole landscape occupancy changes, proximity of infected bank voles to human residences may vary. The probability that vole distribution includes inhabited areas is higher during high-density years. Hence, in addition to a potential change in disease risk stemming from land use or environmental change, e.g. risk of Malaria [1], the spatial risk of NE also varies on a shorter timescale as the range of PUUV-infected bank voles expands and contracts over the course of a cycle. Detailed geographic information on infection in bank voles [39] and NE incidence may also highlight the dynamism of NE risk. If during high vole density years, NE cases were reported from a broader area compared to low vole density years, then countermeasures to reduce future disease risk need to be dynamic rather than locally intensified.

Although NE incidence was strongly related to bank vole density, rainy winters exacerbated human risk. In boreal Sweden, where most NE cases occur; snow conditions during winter are important for small-mammals [16], [18]. Rainy winters, may amongst others, prevent bank voles from accessing food and hiding places [18]. Seeking shelter from adverse weather and snow conditions and from predators, voles may take refuge in human buildings. This phenomenon needs to be investigated in detail to increase our knowledge on the temporal dynamics of NE. As bank voles aggregate indoors, transmission among voles may increase, and indoor viral load becomes elevated, increasing the probability of effective transmission to humans. In Montana in U.S.A, deer mice (*Peromyscus maniculatus*), hosts of Sin Nombre hantavirus had higher sero-prevalence in peri-domestic settings compared to natural settings [40]. Human risk of PUUV infection may be higher than expected based on prevalence measured only in bank voles trapped in natural habitats. Using number of days with average temperature above 0°C instead of number of rainy days in winter to predict NE incidence did not significantly improve model fit, despite the underlying connection between rainy winter days and warmer winter temperatures. This increased our confidence in the genuine role of the rain-on-snow phenomenon in changing bank vole behavior and risk of human infections. To corroborate our findings in relation to weather influence on NE occurrence, direct measurements of snow conditions with and without rain during winter should be undertaken and related to measurements of bank vole activity and numbers indoors or close to human dwellings.

In autumn 2013, bank vole populations were in their increase phase of the cycle; characterized by relatively high densities. We forecast 326 cases in the NE season 2013/2014 using projected autumn bank vole density; with a likely range of 295 to 361 depending on summer bank vole population growth. If the prediction of 326 cases is fulfilled, then the NE season of 2013/2014 would rank 6^th^ highest in number of cases since 1989–1990. However, after this prediction was made, the trapping data from autumn have become available from the long-term trapping area, showing that reality slightly exceeded our bold estimate. Also, in coastal areas, not used in the above predictions, but referred to by Olsson et al. [10], trapping showed that densities there were approximately twice as high as in the inland. Consequently, our forecasts of NE incidence may be conservative, and disease risk could be hence underestimated for 2013/14.

Without any adjustment of the predicted bank vole numbers in autumn, predicted NE-risk was considerably higher in the case of a rainy winter 2013/2014. In this scenario, we predicted 812 NE cases, triple the number predicted in a rain-free winter.

In addition to its cogent fit to NE incidence data, a powerful feature of our prediction model is its simplicity. Our model enables early identification of high risk years, in principle as early as just after trapping in spring, i.e.>6 months ahead the onset of the intense infection period in winter of the ensuing NE season. Predicting NE incidence using observed autumn vole densities, after these become available, would be more accurate. But by that time potentially many NE cases would have already occurred. Early prediction facilitates the implementation of measures to curb NE incidence. From a public health perspective, it is during those years that high preparedness is crucial, and additional warning may also be appropriate later in case of a mild and rainy winter, as this would accentuate infection risk and hence NE incidence.
